# Blood Pressure in Seizures and Epilepsy

**DOI:** 10.3389/fneur.2019.00501

**Published:** 2019-05-14

**Authors:** Robert D. Nass, Kevin G. Hampel, Christian E. Elger, Rainer Surges

**Affiliations:** ^1^Department of Epileptology, University Hospital Bonn, Bonn, Germany; ^2^Department of Neurology, University Hospital La Fe, Valencia, Spain

**Keywords:** hypotension, hypertension, seizure, epilepsy, autonomic nervous system, SUDEP

## Abstract

In this narrative review, we summarize the current knowledge of neurally mediated blood pressure (BP) control and discuss how recently described epilepsy- and seizure-related BP alterations may contribute to premature mortality and sudden unexpected death in epilepsy (SUDEP). Although people with epilepsy display disturbed interictal autonomic function with a shift toward predominant sympathetic activity, prevalence of arterial hypertension is similar in people with and without epilepsy. BP is transiently increased in association with most types of epileptic seizures but may also decrease in some, illustrating that seizure activity can cause both a decrease and increase of BP, probably because of stimulation or inhibition of distinct central autonomic function by epileptic activity that propagates into different neuronal networks of the central autonomic nervous system. The principal regulatory neural loop for short-term BP control is termed baroreflex, mainly involving peripheral sensors and brain stem nuclei. The baroreflex sensitivity (BRS, expressed as change of interbeat interval per change in BP) is intact after focal seizures, whereas BRS is markedly impaired in the early postictal period following generalized convulsive seizures (GCS), possibly due to metabolically mediated muscular hyperemia in skeletal muscles, a massive release of catecholamines and compromised brain stem function. Whilst most SUDEP cases are probably caused by a cardiorespiratory failure during the early postictal period following GCS, a profoundly disturbed BRS may allow a life-threatening drop of systemic BP in the aftermath of GCS, as recently reported in a patient as a plausible cause of SUDEP in a few patients.

## Introduction

People with epilepsy (PwE) have an elevated risk of acute myocardial infarctions and sudden cardiac death as compared to the general population (1–5). Multiple factors increase the likelihood of cardiovascular morbidity and mortality in PwE including detrimental effects of anti-seizure drugs on electrical properties of cardiomyocytes and on circulating blood components (e.g., lipids and related proteins as a major risk factor of coronary artery disease) as well as epilepsy-related negative effects on the autonomic nervous system (ANS) that lead to enhanced sympathetic tone, thereby facilitating cardiac arrhythmias and deregulated control of arterial blood pressure (BP) (6–8). In addition, seizure-related disturbances of cardiac function are frequently observed in association with different seizure types and may be the cause of death in a significant proportion of people with epilepsy (7–9). In this article, we focus on systemic BP, its alterations in PwE and contribution to cardiovascular morbidity, mortality, and possibly sudden unexpected death in epilepsy (SUDEP).

## Neural Control of Blood Pressure

The major determinant of oxygen and metabolite supply of tissues and organs is the blood perfusion which is regionally controlled by activity-dependent mechanisms. The overall perfusion of the body's organs is secured by the systemic BP. Arterial BP is defined as the pressure exerted by the blood on the artery walls (10). By convention it is measured in millimeters of mercury (mm Hg) above the surrounding atmospheric pressure. Mean arterial blood pressure (MAP) is determined by the product of cardiac output (CO) and total peripheral resistance (TPR) (MAP = CO × TPR) while CO is, in turn, the product of stroke volume (SV) and heart rate (HR) (CO = SV × HR) (11). The systemic BP shows a pulsatile profile due to the contraction of the cardiac cycle and the elastic behavior of the artery walls; the maximal pressure during heart contraction is named systolic arterial pressure (SAP), the minimal pressure between two heart contractions as the diastolic arterial pressure (DAP). To secure appropriate energy and oxygen supply, systemic BP is constantly maintained within given limits by regulatory pathways involving the autonomic nervous system. Our current knowledge on the neural control of blood pressure comes from decades of experimental research in animals, clinicopathological correlations in humans, mostly with stroke or epilepsy, electrical stimulation studies in humans with epilepsy and functional imaging studies in humans (12–17).

The peripheral part of ANS consists of the splanchnic nerves, autonomic ganglia and plexus with their adrenergic (sympathetic) or cholinergic (parasympathetic) nerve endings in most organs as well as afferent visceroceptor nerve endings such as baroreceptors in the aortic arch and carotid sinus (measuring BP), volume receptors in the pulmonary veins and atria (measuring blood volume) as well as chemoreceptors e.g., in the lungs' and kidneys' vascular systems (measuring pH, pCO_2_, pO_2_). The central ANS integrates visceromotor, neuroendocrine, pain, and behavioral responses (18). It comprises areas widely dispersed along the neuraxis such as the spinal cord (thoracic intermediolateral column, IML; sacral parasympathetic nuclei), medulla oblongata (nucleus tractus solitarii, NTS; dorsal vagal nucleus, DVN; nucleus ambiguous, NA; ventrolateral medulla oblongata, VLM), pons (parabrachial nucleus, pontine micturition center), mesencephalon (periaqueductal gray), diencephalon (various hypothalamic nuclei, bed nucleus of the stria terminalis, BST; thalamic nuclei), and telencephalon (insular cortex, amygdala, cingulate gyrus, medial prefrontal cortex). In this mini-review, we will focus on structures which are thought to be particularly important in BP control. Visceral afferent fibers reach the NTS in the medulla oblongata, which has multiple connections, most prominently with the neighboring ventrolateral medulla oblongata and other brain stem nuclei that relay to the IML in the thoracic spine as well as the NA and DVN. The caudal ventrolateral medulla oblongata and the rostral ventral medulla oblongata are chief modulators of the sympathetic, preganglionic intermediolateral column neurons in the thoracic spine, whilst the NA and DVN contain the preganglionic neurons of the vagal nerve which innervate the heart and is hence a major regulator of the parasympathetic systems (12, 14, 17).

**Figure 1:**
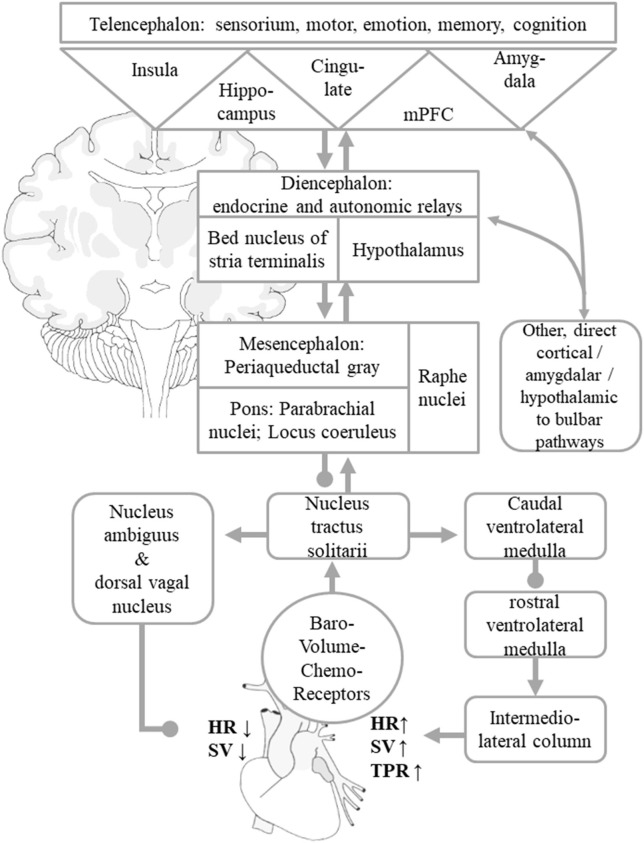
Simplified scheme of central autonomic control of BP homeostasis. Visceral afferents from baro-, volume-, and chemoreceptors reach the NTS, which works as a comparator of information from peripheral visceroaffents and information on behavioral tasks and emotional states signaled by the central autonomic network. These are mediated by indirect and direct connections to the hypothalamus, thalamus, insular cortex, amygdala, cingulate gyrus, and other mPFC areas. These allow adaptations of the neural BP setpoint to given behavioral and emotional states. The NTS asserts control over the NA and DVN, from where parasympathetic efferents mediate a reduction of HR and SV. The NTS regulates sympathetic efferents that originate in the intermediolateral column of the spinal cord by adjusting the caudal and rostral ventrolateral medulla nuclei. The sympathetic system increases total peripheral resistance, HR, and SV. For the sake of clarity, the diagram only shows the indirect “hierarchical” pathways, even though multiple direct pathways from telencephalic and diencephalic visceral control areas to parasympathetic and sympathetic nuclear areas that bypass diencephalic relay centers and the NTS itself exist as well. Arrows indicate predominantly excitatory pathway, dots predominantly inhibitory connections. The figure is adapted from Myers (15), Zanutto et al. (16), and Gianaros and Sheu (19).

The main regulatory loop for short-term BP control is termed baroreflex. It sets HR and SV by an interplay of the sympathetic and parasympathetic system, whereas TPR is predominantly set by the sympathetic activity (Figure 1) (12, 14, 17). If BP drops, for instance in the orthostatic reaction or in the second phase of a Valsalva maneuver, the firing rate of the baroreceptor afferents to the NTS decreases, which will in turn lead to a disinhibition of cardio-acceleratory neurons in the VLM and to an inhibition of cardio-inhibitory neurons in the NA and DVN, thereby increasing HR, SV, and TPR to re-increase BP. If BP rises, for instance during intense muscular effort or the last phase of a Valsalva maneuver, the firing rate of the baroreceptor afferents to the NTS increases, which will in turn inhibit activity of cardio-acceleratory neurons in the VLM and their projections to the IML, which innervate the arterial blood vessels and in turn enhance activity of cardio-inhibitory neurons in the NA and DVN, thereby decreasing HR, SV, and TPR to lower BP again (17). Diencephalic connections of the NTS include thalamic nuclei as well as the hypothalamus and its endocrine regulatory centers. Among the telencephalic connections of NTS, the amygdalar-hippocampal complex and the insular cortex, anterior cingulate gyrus and medial prefrontal area are of particular importance (12–17, 19). These supratentorial centers regulate the “desired” levels of sympathetic and parasympathetic output according to behavioral tasks and emotional states and adjust a neural set point of BP (12–14, 16, 17). The most recognized examples are the activation of the sympathetic nervous system at the same time as the beginning of muscular exercise or in fight or flight situations (16). The central ANS is an integrated, reciprocal, interconnected network, in which isolated parts cover specific, in part lateralized aspects. In Box 1, we give a brief overview of the most widely accepted substrates of the telencephalic autonomic control centers in humans.

Box 1 **Selected Supratentorial Centers of BP Control**

**Insular Cortex**

Situated deeply within the lateral sulcus of the brain and covered by the frontal, temporal, and parietal opercula, the insular cortex has wide ranging, bidirectional connections, e.g., to the dorsal thalamus, frontal, temporal, parietal, cingular, and olfactory cortices as well as the hippocampus and amygdala (20). In the context of BP control, it integrates viscerosensory information from the dorsal thalamus with interoceptive and exteroceptive, memory and cognitive stimuli, including signals of taste, olfaction, temperature perception, auditory processing, vestibular function, pain, emotional experience, empathy, and social cognition. The insular cortex thereby contributes to maintenance of emotional and physiological homeostasis (15, 21). Much of our knowledge on insular function is derived from studies on intracerebral electrical stimulation in PwE (22–24). Electrical stimulation of the insular cortex can have excitatory or inhibitory effects on heart rate, depending on the stimulated insular part and possibly the hemisphere (i.e., the right insula is described to exert more sympathetic and the left insula more parasympathetic activity in some studies) (13, 22, 25–27). Seizures rarely originate in the insula but spread of ictal activity to the insula from adjacent regions is very common. Features of insular seizures include somatosensory, visceral and motor symptoms. They can also mimic frontal lobe, temporal lobe, and parietal lobe seizures (22).

**Medial Prefrontal Cortex**

The medial prefrontal cortex (mPFC) in humans comprises the Brodman areas 32 and 24 in the anterior cingulate region, area 14 in the gyrus rectus and area 25 in the subcallosal area (28, 29). Corresponding regions in rodents, in which much more is known about autonomic mPFC functions are referred to as a infralimbic cortex (area 25) and prelimbic cortex (area 32) (15). They have substantial connections to stria terminalis and raphe nuclei as well as the NTS and posterior hypothalamus, which act as intermediaries to the DVN, NA and IML (15). The mPFC takes part in the integration of visceral sensory and visceral motor signals as well as in the guidance of emotional behavior. The prefrontal cortex receives input from all sensory modalities and uses this information to make the most rewarding decision. Patients with lesions in this area develop either disinhibited or apathetic, dysfunctional behavior and lack the physiological, visceral response to emotional stimuli (29, 30). Seizures originating in the mPFC lead to fearful behavior (31). Decreased mPFC activity in fMRI studies were associated with baroreceptor unloading (32), increased mPFC activity with enhanced HR variability (33). A recent study in PwE undergoing diagnostic video-EEG monitoring using intracranial electrodes revealed that electrical stimulation of the Brodman 25 area led to almost immediate decrease of SAP without affecting DAP or HR, suggesting that this telencephalic region contributes to the BP regulation by selective modulation of cardiac output (34). The corresponding infralimbic region in rodents is considered to drive sympathetic activation (35). The mPFC interacts with the ventral hippocampus in adjusting the HR to exercise (36).

**Amygdala**

The amygdala comprises multiple interconnected nuclei deeply nested in the temporal lobe (37). The amygdala plays an important role in emotional processing, especially in fear and anxiety but also learning and social behavior. The lateral amygdala receives sensory information regarding the external environment from sensory thalamic and sensory cortical afferents. It projects them to the medial, central and basolateral amygdala, which is reciprocally connected with the prefontal cortices, hippocampus and sensory areas. As demonstrated mostly in animal studies, the basolateral amygdala also has indirect connections with the DVN and NA via the bed nucleus of the striatum (BST). The medial amygdala is indirectly connected to the same parasympathetic nuclei via the BST and medial preoptic area (mPOA) of the hypothalamus as well as with the IML neurons in the spinal cord via the posterior hypothalamus. The central amygdala is directly connected to the NTS and VLM and has indirect connection with the NA and DMV through the NTS, BST, mPOA, and parabrachial nuclei, as well as indirect connections with the IML via the lateral hypothalamus, VLM, locus coeruleus, and raphe nuclei (15). In humans, the activation, volume and functional connectivity of the amygdala appear to covary with stressor-evoked BP reactivity and even atherosclerosis. PwE frequently have an increased volume or altered functional connectivity of the amygdala (38).

As outlined above, the short-term BP regulation is secured by the baroreflex; the baroreflex sensitivity (expressed as change of interbeat interval per change in BP) is set to accommodate different states of arousal, stress or physical exertion by the central ANS. Apart from baroreceptor sensitivity, the central ANS is also implicated in the long-term BP control, with the NTS acting as a comparator between peripheral afferents and a setpoint determined by ventral autonomic afferents (16). Dysregulation of this setpoint has an impact on long-term BP control and other effects mediated by the sympathetic system. Furthermore, chronic emotional and psychosocial stress can perpetuate cardiovascular diseases and is a cardiovascular risk factor of similar importance and magnitude as smoking or diabetes (39). This may be partially linked to an increased amygdalar resting state activity, as recently shown in a functional MRI study in apparently healthy adults (40). Apart from chronic stress, acute stress can lead to cardiovascular complications in cases of exaggerated autonomic reactivity during emotional or neurological crisis, the best known of which is the “broken heart syndrome” also known as Takotsubo- or stress-cardiomyopathy (41). Acute stress is a hallmark of seizures and status epilepticus, whilst chronic emotional distress is common in epilepsy (42, 43). Dysfunction of the amygdalar-hippocampal complex itself is one of the most frequent causes of temporal lobe epilepsy, possibly contributing to the elevated rate of cardiovascular diseases in PwE (44). Previous clinical studies have shown an association between epilepsy and an elevated risk of myocardial infarction. In a European study, PwE had an almost 5-fold increased risk for myocardial infarction and poorer prognosis thereafter, independent of age, sex, location, and classic cardiovascular risk factors (3). These findings were largely replicated in a Chinese study showing that people with newly diagnosed epilepsy were 4–5 times more likely to acquire or die of a heart disease and stroke than age-matched controls, especially if enzyme inducing anti-seizure drugs were used (45). Another recent US-American study confirmed the elevated risk of myocardial infarction in PwE (46). In this context, we want to stress that a few cases labeled as SUDEP and in whom further diagnostics or subsequent postmortem were not performed, myocardial infarction may underlie the sudden death. However, if cardiac diagnostics in the acute phase or postmortem are done and display signs of acute myocardial infarction, the death is—by definition—not due to SUDEP (47).

## Seizure-Related Changes in Cardiac Autonomic Function

Besides the above-mentioned chronically abnormal activity in brain regions involved in the regulation of BP control, seizures themselves often exert acute effects on various functions of the autonomic nervous system. These include gastrointestinal (spitting, nausea, vomiting, defecation) and other vegetative reactions (piloerection, urination, skin flush etc.) as well as alterations of respiratory (tachypnea, hypopnea, apnea) and cardiac function (e.g., tachycardia, bradycardia) (48).

Sympathetic outflow is commonly enhanced during seizures, as shown by elevated levels of circulating catecholamines (49, 50), increased HR, QT-shortening, elevated electrodermal activity or reduced HR variability (51, 52). About 80% of focal seizures go along with ictal sinus tachycardia (which increases CO), whilst cardiac arrhythmias such as atrial fibrillation or ventricular tachycardias are very rare (53). Ictal bradycardia is less common than tachycardia and occurs mostly in temporal lobe seizures. Ictal asystole was detected in about 0.3% of focal seizures (FS) recorded in video-EEG monitoring units, notably with a recurrence risk of ~40% (54, 55). The mechanisms underlying ictal bradycardia and asystole may include an acute, directly seizure-related dysregulation of parasympathetic networks in the amygdala, cingulate gyrus, and insular cortex or the activation of the physiologic vagal reflex pathway (44, 55). Ictal asystole shortens and terminates the seizure activity because of global cerebral hypoperfusion, possibly preventing the evolution to generalized convulsive seizures (GCS) (56, 57). The related BP drop, however, may also cause syncope with loss of muscle tone and risk of falls and injuries (57–59). All reported episodes with ictal asystole and bradycardias were self-limited, suggesting that ictal asystole is usually not linked to SUDEP (54). In view of the high recurrence risk and the associated danger of falls and injuries, however, the implantation of a cardiac pacemaker should be considered in affected patients in whom full seizure control cannot be achieved (55, 59–61). In contrast to ictal asystole, postictal asystole appears to be less frequent and was exclusively reported to occur in the early phase after GCS [including focal to bilateral tonic-clonic seizures (FBTCS) and generalized tonic-clonic seizures]. Postictal asystole is commonly secondary and caused by severe hypoxemia (which suppresses heart activity) which, in turn, is due to postictal central apnea (62). The mechanisms leading to postictal apnea are not well understood but may be related to postictal generalized suppression of brain activity and a direct depression or increased inhibition of respiratory centers in the brainstem. The fatal cascade consisting of GCS → postictal apnea → hypoxemia → terminal asystole is likely to be the commonest cause of SUDEP and may be reversed by immediate cardiopulmonary resuscitation (54, 62).

## Seizure-Related Changes in Blood Pressure

While the effects of seizures on HR were extensively studied and recently reviewed (44), seizure-related alterations of BP are less well investigated, mainly because of methodical issues. For instance, studies with intermittent BP monitoring using conventional cuffs attached to the upper arm do not allow capturing rapid changes and the peri-ictal time course of BP (63, 64). Intraarterial BP recordings were anecdotally reported during epileptic seizures but are not suited for systematic larger scale studies (65). Non-invasive methods to continuously measure beat-to-beat BP are available nowadays and allow recording of the time course of peri-ictal BP changes but may be compromised by movement-related artifacts (34, 66–70).

### Blood Pressure During Focal Seizures

**Figure 2:**
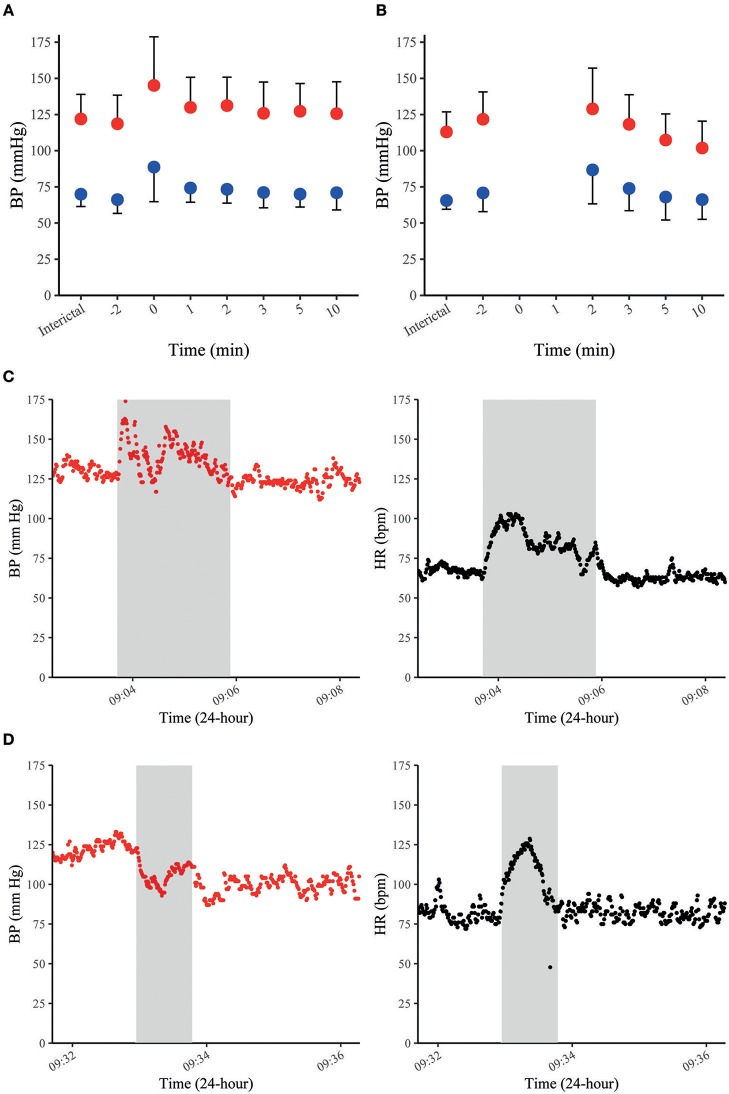
BP in FS and FBTCS. Summary graphs of seizure-related SAP (in red) and DAP (in blue) at different time points in 35 FS of 28 patients **(A)**. Summary graphs of seizure-related SAP (in red) and DAP (in blue) at different time points in 10 FBTCS of 9 patients **(B)**. The x-axis represents different peri-ictal time points (e.g., −2 equals two min prior to the seizure-onset, 0 indicates the time of the seizure). The point charts represent mean ± SD. Examples of time-course of SAP and HR during FS in a patient with concomitant increase of SAP and HR **(C)** and a decrease of SAP and increase of HR in another patient **(D)**. The gray boxes indicate the duration of individual seizures in each figure. Data were previously published in Hampel et al. (70).

In our recent study with continuous non-invasive BP recordings in 37 patients with focal epilepsy undergoing video-EEG monitoring, MAP, SAP, and DAP increased by 20–30% on average during 35 FS and returned to baseline within 10 min after seizures cessation (Figure 2A) (70). Peri-ictal alterations of BP had a similar time course as the concomitant increase in HR and did not depend on oxygen saturation. FS with impaired awareness showed a stronger increase in BP than those without impaired awareness (70). Notably, peri-ictal BP modulation was stereotypic in those patients with recordings of more than one seizure of the same type. The most frequent pattern was a concomitant increase of BP and HR, which is in line with previously published case reports (Figure 2C) (69, 71). In some patients with FS, however, BP decreased whilst HR increased (Figure 2D) (70). Jaychandran and colleagues also found, on average, a seizure-related increase in BP in 42 patients with 57 FS (72). They reported that ictal hypertension (defined as SAP>140 mm Hg and/or DAP>90 mm Hg) was observed in 26.3% of the patients, whereas ictal hypotension (defined as SAP < 90 mm Hg and/or DAP < 60 mm Hg) occurred in 8.7%.

The mechanisms leading to seizure-related BP alterations are unclear, but probably involve stimulation or inhibition of central autonomic function by epileptic activity that propagates into neuronal networks of the central ANS. For example, electrical stimulation of insular and thalamic areas as well as basal ganglia in humans can increase both BP and HR (27, 73). In addition, FS may also increase HR and BP through a release of catecholamines via stimulation of adrenergic receptors in heart and blood vessels (49, 50, 71). Surprisingly, a seizure-related decrease of BP was accompanied by an increase in HR in a subgroup of patients (Figure 2D) (70). This pattern suggests that the pathways modulating BP and HR involve distinct brain regions. This assumption is further supported by the finding that 3 patients with implanted depth electrodes showed a significant drop of SAP upon electrical stimulation of the mPFCs Brodmann area 25 without apparent changes in HR or DAP (35). Thus, Brodmann area 25 is possibly a symptomatogenic zone of cardiac contractility (and SV) leading to ictal hypotension.

### Blood Pressure During Generalized Convulsive Seizures

Data on BP during GCS are scarce because seizure-related movements usually prevent reliable BP measurements throughout the tonic–clonic phase (70, 72). According to anecdotal reports, BP appears to have two distinct patterns during the *ictal* phase of GCS. Pattern 1 was observed in two GCS of two patients and consisted of a concomitant increase of BP and HR (65, 68). Pattern 2 was documented in two GCS of two other patients and displayed an initial BP increase which was rapidly followed by a considerable drop in BP (65). Pattern 1 could also be explained by a seizure-induced stimulation of the sympathetic branch of the central nervous network and catecholamine release (49). Pattern 2 with an early drop of BP may be due to central autonomic effects rather than a Valsalva-mediated reflex, as it has also been described in muscle-relaxed and highly oxygenated depressive patients during electroconvulsive therapy (74). In the *postictal* phase, BP returns to baseline within a few minutes after seizure termination or, if BP has decreased during the ictal phase, it increases again and then returns to baseline within a few minutes (65, 70). In our study, postictal MAP, SAP, and DAP were slightly elevated and then dropped to baseline or even below pre-ictal values 2 min after seizure cessation (Figure 2B) (70). In the early postictal phase, SAP was less elevated than MAP and DAP, but decreased even stronger within some minutes after seizure cessation. In contrast to BP, HR was strongly elevated 2 min after seizures termination and remained elevated 10 min after seizure cessation. This opposite time course of BP and HR is possibly caused by an immediate muscular hyperemia (that frequently follows exercise of skeletal muscles) finally leading to a decreased TPR and a subsequent drop in systemic BP (75). An alternative explanation is that cardiac contractility is impaired postictally, causing a relatively selective decrease in SV and SAP. Although systematic studies on heart function in the early postictal period are lacking to confirm this assumption, cases of seizure-related stress cardiomyopathy and even frank Takotsubo cardiomyopathy with ventricular fibrillation have been reported, suggesting that especially GCS may alter cardiac contractility (76, 77).

A drop in systemic BP should be counteracted by an increase of HR via the arterial baroreflex (Figure 1) which, in turn, may be compromised by seizure-related alterations of the reflex loop. Indeed, the baroreflex sensitivity (BRS) was recently shown to be markedly impaired in the early postictal period following 7 FBTCS in 7 patients, whereas BRS was intact after 19 FS in 19 patients (78). These findings were largely replicated in a study by Esmaeili et al. including 9 FBTCS and 14 FS of 18 patients (79). The apparent impairment of BRS in the aftermaths of FBTCS is possibly due to metabolically mediated muscular hyperemia in skeletal muscles following the generalized tonic-clonic convulsions (which overdrives neutrally-mediated sympathetic effects) on the one hand and the massive release of catecholamines with subsequent acceleration of HR on the other hand. Alternatively, exhaustion or suppression of neuronal activity after FBTCS may compromise brain stem function including the networks in the caudal VLM, the rostral ventral medulla, and the NA (68, 80). For instance, FBTCS are commonly followed by a postictal generalized electroencephalographic suppression (PGES) (81, 82) and in one patient, postictal hypotension was observed in association with PGES (68). Opposite to this assumption, however, postictal changes of BP and BRS were not related to occurrence or duration of PGES (70, 78, 79). Altogether, these results must be taken with caution and larger-scale studies are needed to confirm this hypothesis.

### Clinical Implications of Seizure-Related Alterations of Blood Pressure and Baroreflex Sensitivity

Most SUDEP cases are probably caused by a cardiorespiratory failure during the early postictal period following GCS (62). Current data suggest that BP changes in association with FS are moderate and BRS is not significantly altered (70, 72). In GCS, however, BRS is markedly impaired postictally on the one hand and postictal BP appears to return rapidly to or below baseline levels (70, 78, 79). A recent case report even described a life-threatening drop of systemic BP with a MAP of 40 mm Hg in the aftermath of a GCS (68). Such a dramatic hypotension in combination with an impaired BRS is likely to favor the fatal SUDEP cascade, as an insufficient compensatory baroreflex response to decreased BP may compromise systemic or cerebral blood supply and cause significant hypoxemia of the organs (83). When exceeding given thresholds, the deprivation of oxygen and metabolites could result in irreversible tissue damage or dysfunction, facilitating in turn mechanisms ultimately leading to SUDEP.

## Interictal Alterations of Blood Pressure and Baroreflex in Epilepsy

According to previous surveys, the prevalence of arterial hypertension is similar in PwE as compared to the general population (84). This is in line with smaller scope studies reporting similar interictal BP values in people with FBTCS and healthy controls (85) as well as in individuals with epilepsy who later died of SUDEP and two matched control groups with and without epilepsy (86). The authors of the latter study found, however, that DAP tended to be higher in SUDEP patients, suggesting that the sympathetic tone is elevated in this patient group (86). In fact, subtle signs of cardiovascular autonomic dysfunction such as altered HR variability (HRV) at rest (87) or in response to orthostasis and other autonomic tests are common in PwE, possibly augmented by anti-seizure drugs such as carbamazepine (88). For instance, attenuated HRV, which is an established risk factor for cardiovascular morbidity and mortality, is significantly decreased in PwE, indicating a shift of autonomic function toward a predominant sympathetic activity and lower vagal activity. This sympathovagal imbalance may be further reinforced due to the effect of anti-seizure drugs (88, 89) and during phases of sleep related apnea, both in people with focal and generalized epilepsy syndromes (90, 91).

Furthermore, interictal BRS was shown to be impaired in people with temporal lobe epilepsy (92) and reflex epilepsy (93), adding to the notion that PwE may be more vulnerable in regard to cardiovascular diseases due to autonomic imbalances such as alterations of baroreflex function, which might in fact be more common than assumed.

In PwE, interictal alterations of autonomic function and BP homeostasis may be at least partially due to acute or chronic side-effects of anti-seizure drugs. For instance, rapid intravenous application of phenytoin and sedatives such as barbiturates, benzodiazepines, and anesthetic agents are known to lower BP or to induce hypotension (94). These acute effects are likely to be induced by inhibition of voltage-gated sodium and calcium channels with subsequent decrease of cardiac contractility and SV [for review see e.g., (95)]. Probably the most common effects of anti-seizure agents on BP are of indirect nature and related to weight gain [e.g., upon intake of valproic acid, gabapentin, and pregabalin (6)] and detrimental effects on circulating cardiovascular risk factors such as dyslipidemia and hyperhomocysteinemia mostly caused by enzyme-inducing anti-seizure drugs (e.g., carbamazepine, phenobarbital, phenytoin) which in turn may lead to atherosclerosis with decreased blood vessel flexibility and reactivity (96–98). A minor subgroup of cases with sudden death may be explainable due to genetic overlaps with genetically caused cardiac arrhythmias and epilepsy, e.g., by mutations in potassium channel genes KCNQ1 and KCNH2 or sodium channel genes SCN5A (99).

## Conclusions

Systemic BP is permanently monitored and maintained within given limits by the baroreflex loop which, in turn, is modulated by supratentorial and cortical neuronal networks involved in the central ANS. Although PwE display altered interictal autonomic function with a shift toward predominant sympathetic activity and impaired BRS, prevalence of arterial hypertension appears to be similar in PwE as compared to the general population. BP is transiently increased in association with most types of epileptic seizures but may also decrease in some. Postictal arterial hypotension is facilitated by metabolically mediated muscular hyperemia in skeletal muscles and an impaired BRS following GCS, facilitating insufficient blood supply and possibly life-threatening hypoperfusion of body organs as a plausible cause of SUDEP in some cases.

## Author Contributions

RN drafted the manuscript and created Figure 1. KH has contributed to the writing of the manuscript and created Figure 2. CE has critically revised the manuscript for important intellectual content. RS has conceived and revised the manuscript.

### Conflict of Interest Statement

RN has received fees as a speaker and consultant from Eisai. KH has received support from Cyberonics and Eisai. CE has received support from UCB Pharma, Desitin, and Pfizer. RS has received fees as speaker or consultant from Bial, Cyberonics, Desitin, Eisai, LivaNova, Novartis, and UCB Pharma. The funders were not involved in the study design, collection, analysis, interpretation of data, the writing of this article or the decision to submit it for publication.

## Abbreviations

ANS: autonomic nervous system
BP: blood pressure
BRS: baroreflex sensitivity
BST: bed nucleus of the stria terminalis
CO: cardiac output
DAP: diastolic arterial pressure
DVN: dorsal vagal nucleus
FS: focal seizure(s)
FBTCS: focal to bilateral tonic-clonic seizure(s)
GCS: generalized convulsive seizure(s)
HR: heart rate
IML: intermediolateral column
MAP: mean arterial blood pressure
mm Hg: millimeters of mercury
mPFC: medial prefrontal cortex
mPOA: medial preoptic area
NA: nucleus ambiguous
NTS: nucleus tractus solitarii
pCO_2_: partial pressure of carbon dioxide
PGES: postictal generalized electroencephalographic suppression
pO_2_: partial pressure of oxygen
PwE: people with epilepsy
SAP: systolic arterial pressure
SUDEP: sudden unexpected death in epilepsy
SV: stroke volume
TPR: total peripheral resistance
VLM: ventrolateral medulla oblongata.
